# Relationship Between Performance of Carob Moth, *Ectomyelois ceratoniae* Zeller (Lepidoptera: Pyralidae) and Phytochemical Metabolites in Various Pomegranate Cultivars

**DOI:** 10.3389/fphys.2019.01425

**Published:** 2019-11-19

**Authors:** Zahra Abedi, Ali Golizadeh, Mahmoud Soufbaf, Mahdi Hassanpour, Ali Jafari-Nodoushan, Hamid-Reza Akhavan

**Affiliations:** ^1^Department of Plant Protection, Faculty of Agriculture and Natural Resources, University of Mohaghegh Ardabili, Ardabil, Iran; ^2^Department of Plant Protection, Nuclear Agriculture Research School, Nuclear Science and Technology Research Institute, Karaj, Iran; ^3^Agriculture and Natural Resources Research Center of Yazd, Yazd, Iran; ^4^Department of Food Science and Technology, Faculty of Agriculture, Shahid Bahonar University of Kerman, Kerman, Iran

**Keywords:** *Ectomyelois ceratoniae*, pomegranate, feeding response, physiological characteristics, demographic parameters, phytochemical metabolites

## Abstract

The carob moth, *Ectomyelois ceratoniae* Zeller (Lepidoptera: Pyralidae), is the main pest of pomegranate, *Punica granatum* L., in Iran and many parts of the world. In this study, the effects of 11 commercial cultivars of pomegranate (Aban-Mahi, Esfahani-Daneghermez, Gabri, Gorche-Tafti, Malase-Danesyah, Malase-Yazdi, Shahvare-Daneghermez, Shahvare-Danesefid, Tabolarze-Mehrmahi, Tafti, and Toghe-Gardan) were evaluated on life history variables, nutritional performance, and energy reserves of *E. ceratoniae* under the following laboratory conditions: 30 ± 1°C, 60 ± 5% RH, and a photoperiod of 14:10 (L:D) hours. In addition, biochemical characteristics of the tested cultivars were assessed in order to understand any possible correlation between important demographic parameters and nutritional properties with biochemical features of pomegranate juice. Our research showed that various pomegranate cultivars have significant effects on life history, demographical parameters, nutritional indices, and energy reserves of *E. ceratoniae*. The shortest development time was observed on Shahvare-Danesefid cultivar and the longest was on Esfahani-Daneghermez and Malase-Danesyah cultivars. The highest intrinsic rate of increase (*r*_*m*_) was observed on Shahvare-Danesefid and the lowest was on Esfahani-Daneghermez. Six major anthocyanin compounds were detected in juice of various pomegranate cultivars. Significant positive or negative correlations were observed between life history variables and nutritional characteristics with biochemical traits of pomegranate cultivars. The results indicated that Shahvare-Danesefid was a relatively susceptible pomegranate cultivar and Esfahani-Daneghermez was the least appropriate (most resistant) cultivar for feeding of *E. ceratoniae*, which could be useful in the development of integrated pest management strategies for this pest.

## Introduction

The carob moth, *Ectomyelois ceratoniae* Zeller (Lepidoptera: Pyralidae), has been recognized as one of the most destructive insect pests of pomegranate and other fruits such as date, almond, carob, nuts, walnut, fig, and pistachio, with a broad distribution in Iran and many tropical and subtropical regions (Warner et al., 1990; Ranjbar et al., 2011; Kishani-Farahani et al., 2012; Soufbaf et al., 2017). Pomegranate, *Punica granatum* L. (Ponicaceae), is the main host of the carob moth *E. ceratoniae* in Iran. The larvae feed inside the fruit, resulting in contamination with saprophytic fungi and causing great damage during the cropping season and after harvest, conditions that make the fruit unfit for human consumption (Shakeri, 2004).

The use of resistant cultivars could be an effective approach in integrated pest management (IPM) strategies targeting the carob moth (*E. ceratoniae*) in pomegranate orchards (Ramzi et al., 2014; Sobhani et al., 2015). Host plant resistance is an alternative for pest management as it is both economically and environmentally acceptable (Golizadeh et al., 2009; Soufbaf et al., 2010; Golizadeh and Abedi, 2016). Evaluating the resistance of various cultivars and crop species to pests may offer useful information about their suitability or unsuitability for the target pest species (Tsai and Wang, 2001). Host plant resistance to insects can also be manipulated using chemical elicitation of secondary metabolites, which can confer resistance to insects (War et al., 2012). Proper use of resistant cultivars in pest management strategies requires knowledge of life table parameters and biological variables of pests (Nawrot et al., 2010; Golizadeh and Abedi, 2017; Gvozdenac et al., 2018; Karimi-Pormehr et al., 2018).

Previously, Norouzi et al. (2008) studied population growth traits of the carob moth fed with different fruits like pomegranate, pistachio, fig, and date; their results showed that the reproductive output of *E. ceratoniae* on pomegranate and pistachio was higher than that on fig and date. Zare et al. (2013) investigated the biological characteristics of this pest on three pomegranate cultivars and indicated that Malase-danesyah cultivar was the least susceptible host for *A. ceratoniae*. Mortazavi et al. (2016) evaluated the effects of four diets including pistachio, pomegranate, semi-artificial and artificial diets on this pest, measured some of the biological variables and reported that pomegranate was the most suitable host for *E. ceratoniae*.

Various defense characteristics of host plants affect the fitness of herbivorous insects (Harvey et al., 2007; Nouri-Ganbalani et al., 2018). Secondary plant chemicals act as feeding inhibitors, deterrents, repellents, and antidigestive compounds that interfere with herbivore physiology and reduce its developmental and survival rates as well as its potential fecundity (War et al., 2011; Nikooei et al., 2015). Pomegranate juice is a potential source of secondary metabolites such as anthocyanins, ellagic acid, phytoestrogenic flavonoids, organic acids, tannins, and antioxidants (Tezcan et al., 2009; Fischer et al., 2011). Anthocyanins are polyphenolic compounds (flavonoids) responsible for the blue, red, and purple colors of most fruits and flowers, which are major compounds of pomegranate juices (Du et al., 1975; Alighourchi et al., 2008; Turfan et al., 2011; Mirsaeedghazi et al., 2014; Akhavan et al., 2015). Important anthocyanins in pomegranate juice are cyanidin 3-glucoside, cyanidin 3,5-diglucoside, delphinidin 3-glucoside, delphinidin 3,5 diglucoside, pelargonidin 3-glucoside, and pelargonidin 3,5-diglucoside (Alighourchi et al., 2008; Turfan et al., 2011; Mirsaeedghazi et al., 2014).

Pomegranate is native to Iran and northern India, however nowadays it is widely cultivated in subtropical regions of the world (Sarkhosh et al., 2009). Iran is the first producer of pomegranate with 6400 ha of pomegranate orchard and total production of 700,000 ton annually. The high quality of pomegranates in Iran has made it an important export commodity and some commercial cultivars have been exported from Iran to other parts of the world (Zare et al., 2013; Ashtari et al., 2019). Therefore, the findings of this study could be used wherever these cultivars are cultivated.

The aim of the present study was to determine whether the life history and nutritional traits of *E. ceratoniae* vary as a function of the commercial pomegranate cultivar used to rear larvae, using a two-sex life table method. In parallel, we wanted to examine how the biochemical properties and/or nutritional quality of the cultivars we tested can affect life history variables and the nutritional performance of *E. ceratoniae*. To this end, we quantified some pomegranate compounds, especially major anthocyanins in the juice of various pomegranate varieties, and examined the relationship between their levels and selected biological variables in the pest; prior to this study, no published information was available on the correlation between such variables in *E. ceratoniae* and plants contents in major anthocyanins and other biochemical compounds of pomegranate juice. Our findings will inform the development of novel carob moth management approaches, including the use of resistant cultivars to reduce pest damage in orchard systems.

## Materials and Methods

### Pomegranate Cultivars Tested

Fruits of 11 pomegranate (*P. granatum*) cultivars, including Aban-Mahi, Esfahani-Daneghermez, Gabri, Gorche-Tafti, Malase-Danesyah, Malase-Yazdi, Shahvare-Daneghermez, Shahvare-Danesefid, Tabolarze-Mehrmahi, Tafti, and Toghe-Gardan, which are commercially important in Iran, were selected from mature trees grown in Agricultural and Natural Resources Research Center (ANRRC) of Yazd, Iran (31° 54′ 54′′ N, 54° 16′ 37′′ E). Commercially ripe fresh fruits were randomly selected and harvested from different mature trees to represent the population of the plantation during September 2016. All cultivars were grown within the same geographical zone, using similar agronomic practices. The fruits were transported by a ventilated car to the laboratory, where pomegranates with defects (sunburns, cracks, cuts, and bruises in peel) were discarded. Approximately 10 kg (*n* = 60) of pomegranate fruit was sampled for each cultivar. Five pomegranates of each cultivar were used for juice extraction, which was kept frozen at −80°C until analysis. The remainder of the fruits was placed in paper bags, stored in refrigerators at 2°C and then used for feeding of larvae.

### Insect Rearing

Larvae used to establish the colony of *E. ceratoniae* were collected in orchards at the Agricultural and Natural Resources Research Center, Yazd (Iran), between August and October 2016. The pomegranate fruits infested with carob moth larvae were maintained in a growth chamber at 30 ± 1°C, relative humidity of 60 ± 5%, and under a photoperiod of 14 h:10 h (L:D). New adults that emerged from infested fruits were caught with an electric aspirator and transferred to the mating cages (50 cm × 50 cm × 100 cm) for 24 h (Navarro et al., 1986). Adults were fed with a 10% honey-water solution. After mating and flying in mating cages, 20 pairs of adult moths were placed in a transparent cylindrical plexiglass container (30 cm × 18 cm) with two holes in the cap covered by organza mesh, and the egg-loaded organza was replaced daily with a new one until nearly all the moths died. The newly laid eggs were separately transferred on 11 pomegranate cultivars. Larvae were reared on each pomegranate cultivar in plastic containers (diameter: 20 cm, depth: 6 cm) with a hole covered by a mesh net for ventilation and maintained under the abovementioned standard rearing conditions. Before initiating the experimental work, insects on each pomegranate cultivar were reared for two generations on the same cultivar, and then eggs from the third generation were used to conduct the experiments.

### Life History Variables and Body Weight in Immature Stages

The experiments on each pomegranate cultivar started using same-aged eggs (within 24 h after oviposition) laid by females reared as larvae on the same cultivar. Sixty eggs of *E. ceratoniae* were individually transferred into Petri dishes (diameter 9 cm, depth 2 cm). In order to ventilation, the middle part of the lid of Petri dish was cut in a circle (diameter 3 cm) and then covered with a cloth mesh. The Petri dishes contained a part of pomegranate fruit for feeding of larvae. The fruit pieces were replaced with fresh ones every 2 days during the larval development. The fifth instar larvae of *E. ceratoniae* reared on each cultivar were weighed separately 24 h after their emergence. The Petri dishes were checked daily, and the duration of immature stages (egg, larval, pre-pupal, and pupal stages), and immature survival rate were recorded. Pupal weight of *E. ceratoniae* was measured within 24 h of pupation on each pomegranate cultivar (Sartorius AG Germany GCA803S, *d* = 0.001 ct). All experiments on pomegranate cultivars were carried out under the following laboratory conditions: 30 ± 1°C, relative humidity of 60 ± 5%, and a photoperiod of 14:10 (L:D).

### Life History Variables in Adults

After emergence of adult moths, a pair of female and male moths was transferred to the same mating cages as described above and maintained for 24 h. Then, each pair was placed in a transparent plastic tube fitted with mesh lids (6 cm diameter and 15 cm depth). Experimental tubes were checked daily and the number of *E. ceratoniae* eggs deposited in each tube was recorded. In this way, each pair was daily transferred to a new tube, the number of eggs laid by adults was counted, and the experiments were continued up to the death of the last adult female and male moth. To supply a source of carbohydrate for adult feeding, a small cotton wick soaked in 10% honey solution was inserted into the egg- laying container. In this experiment, adult preoviposition period (APOP: the time between the emergence of an adult female and the start of its oviposition), total preoviposition period (TPOP: the duration from egg to first oviposition), oviposition period, fecundity (eggs laid during the reproductive period), and adult longevity were recorded. Resulting data were analyzed based on the age-stage, two-sex life table model developed by Chi and Liu (1985) and Chi (1988).

### Nutritional Indices and Quantitative Analysis of Stored Macromolecules in Larvae

The gravimetric method described by Waldbauer (1968) was used to determine the nutritional responses (larval weight, food consumed, feces produced, and weight gain) of *E. ceratoniae* fourth-instar larvae on 11 pomegranate cultivars (40 replicates for each cultivar). Nutritional indices were evaluated on the basis of dry weight. Fourth-instar larvae were placed on fruit of each pomegranate cultivar after weighing and transfer into Petri dishes (9 cm in diameter, depth 2 cm). Larval weight was recorded daily, before and after feeding, for a 1-week experimental period. The fresh food provided, the remaining food, and feces at the end of each experiment were weighed daily. The quantity of ingested food was calculated by subtracting the weight of remaining food at the end of each experiment from the weight of fresh food at the beginning. To obtain the dry weight percentage of the food and larvae, 20 specimens of food and larvae for each cultivar were weighed, oven-dried at 60°C for 48 h, and then weighed again. Nutritional indices of larval *E. ceratoniae* were calculated using the Waldbauer (1968) as follows: efficiency of conversion of ingested food (ECI) = P/E; efficiency of conversion of digested food (ECD) = P/E-F; relative consumption rate (RCR) = E/A^∗^T; relative growth rate (RGR) = P/A^∗^T; where A = mean dry weight of larvae over the feeding period (g), E = dry weight of food consumed (g), F = dry weight of feces produced (g), P = dry weight gain of larvae (g), and T = duration of feeding period (day).

To measure the energy content of *E. ceratoniae* larvae (protein and glycogen contents), we used the whole body of fourth-instar larvae feeding on each pomegranate cultivar. All assays were replicated three times.

Total protein concentration of individual larvae was assessed using the Bradford (1976), bovine serum albumin (BSA) as a standard.

Glycogen content was assayed using the anthrone reagent as described (Yuval et al., 1998) and whole body lipid of *E. ceratoniae* larva was extracted according to the Van Handel (1985) method.

### Biochemical Traits of Pomegranate Cultivars

Biochemical traits of pomegranate were assessed to examine the correlation between the main life-table parameters, nutritional indices, and energy reserves of *E. ceratoniae*, on the one hand, and biochemical compounds founds in various pomegranate cultivars. All assays (each pomegranate cultivar) were done in three replicates. All biochemical metabolites of pomegranate were measured using pomegranate juice. Before juice extraction, fruits of 11 pomegranate cultivars (five from each cultivar) were washed in cold tap water and dried, and the damaged pomegranates were discarded. The juicy sacs from fruit pericarp were separated by hand and the juices were obtained using a manual juicer via pressing the arils. The juice samples (200 mL) were immediately centrifuged (10,000 rpm for 2 min at 4°C), divided into small vials and frozen at −80°C until analysis (Alighourchi et al., 2013).

### Anthocyanin Analysis

Delphinidin 3,5-diglucoside, delphinidin 3-diglucoside, cyanidin 3,5-diglucoside, cyanidin 3-glucoside, pelargonidin 3,5-diglucoside, and pelargonidin 3-glucoside standards were purchased from Apin Chemicals, Co., Ltd. (Oxon, United Kingdom). Anthocyanins in juices were determined by high-performance liquid chromatography (HPLC) using an HPLC system equipped with an Empower software, a pump (Waters 600), a Rheodyne 7125i six-way injector with 20 μL sample loop, and a UV–Vis detector (Waters model 2487). Twenty microliters of purified juice was injected onto the HPLC column. Calculation of concentrations was based on the external standard method and anthocyanins were identified by comparison of their retention times with those of pure standards.

### Total Protein Concentration Measurements

Protein content of pomegranate cultivars we tested was determined according to the Bradford (1976) using BSA as a standard.

### Carbohydrate Content Determination

The carbohydrate content was assayed using the Anthrone reagent (0.05% in sulfuric acid) (Bemani et al., 2012).

### Condensed Tannin (CT) Content Determination

Condensed tannin content reactive to Sulphosphovanillin reagent (orthophosphoric acid 0.6% aqueous vanillin solution 4:1 v/v) was analyzed according to Tanner and Brunner (1979). Quantification of condensed tannin content was carried out using calibration curves of catechin as an external standard.

### Total Monomeric Anthocyanin Pigment Content (TMAC)

Total anthocyanin contents were measured by pH differential method using two buffer systems: potassium chloride, pH 1.0 (0.025M) and sodium acetate, pH 4.5 (0.4M) (Wrolstad et al., 2005). Results were expressed as mg of cyaniding-3-glucoside per 100 mL of pomegranate juice.

### Total Phenolic Content (TPC)

Total phenolic content was determined using Folin–Ciocalteu method described by Tezcan et al. (2009). The result was expressed as milligram of gallic acid equivalent per 100 mL of pomegranate juice.

### Flavonoid Content Determination

Total flavonoid content was measured by aluminum chloride colorimetric assay (Jia et al., 1999). Total flavonoid content was expressed as milligram catechin equivalents per one milliliter of extract.

### Antioxidant Activity Determination

Evaluation of antioxidant activity was done based on radical scavenging properties of the juice using 2,2-diphenyl-1-picrylhydrazyl (DPPH) method (Tezcan et al., 2009).

### Titrable Acidity (TA), pH, and Soluble Solids Content

Total titrable acidity was determined by titration to pH = 8.1 with 0.1M NaOH solution and expressed as grams citric acid per liter of juice. The pH measurements were performed using a digital pH meter at 20°C. Total soluble solid content (SSC) was determined with a digital refractometer. Results were reported as°Brix at 20°C (Alighourchi et al., 2008).

### Statistical Analysis

Before analysis, all data were examined for normality using Kolmogorov–Smirnov test by SPSS v. 16.0 statistical program (SPSS Inc, 2007). The life table parameters including the intrinsic rate of increase (*r*_*m*_), gross reproductive rate (*GRR*), net reproductive rate (*R*_0_), mean generation time (*T*), and finite rate of increase (λ) were analyzed according to the age-stage, two-sex life table using TWOSEX-MSChart (Chi, 2016). The variability of life table parameters was estimated in bootstrap procedure and the bootstrap values on 11 pomegranate cultivars were compared using paired bootstrap test (*P* < 0.05) (Chi, 2016). The developmental time, immature survival rate, and fecundity were used for calculation of age-specific survivorship (*l*_*x*_) and age-specific fecundity (*m*_*x*_) for both male and female (Chi and Su, 2006). Additionally, weights of fifth instar larvae and pupae, nutritional indices, energy reserves of *E. ceratoniae* and biochemical traits of various pomegranate cultivars were analyzed by one-way ANOVA with mean separation at 5% level of significance by Tukey test (SAS Institute, 2002). Correlation between some important demographic parameters, nutritional indices, and storage macromolecule amounts of *E. ceratoniae* with biochemical compounds of various pomegranate cultivars was evaluated through Pearson’s correlation test. A dendrogram of pomegranate cultivars based on life table parameters, nutritional indices, and storage macromolecules of *E. ceratoniae* on tested pomegranate cultivars was constructed by Ward’s method using SPSS.

## Results

### Immature Life History Variables and Body Weight

Developmental time of *E. ceratoniae* on various pomegranate cultivars is given in Table 1. There were significant differences in mean incubation periods among the pomegranate cultivars. The longest egg incubation period was on the Esfahani-Daneghermez and Malase-Danesyah cultivars, while the shortest was observed on the Toghe-Gardan cultivar. The longest larval period was seen on the Esfahani-Daneghermez cultivar and the shortest on the Shahvare-Danesefid cultivar. The pre-pupal period of *E. ceratoniae* on the Shahvare-Danesefid cultivar was significantly shorter than on the other cultivars except for Malase-Yazdi. The longest pupal period was observed on the Esfahani-Daneghermez and Malase-Danesyah cultivars, whereas a shorter period was observed on the Toghe-Gardan cultivar. The shortest developmental time from egg to adult emergence was on the Shahvare-Danesefid cultivar (36.41 ± 0.31 days) and the longest was on the Esfahani-Daneghermez (41.80 ± 0.26 days) and Malase-Danesyah (41.46 ± 0.28 days) cultivars. The immature survival rate ranged from 0.68 ± 0.06 to 0.87 ± 0.05 with the lowest and highest survival rates observed on Esfahani-Daneghermez and Shahvare-Danesefid cultivars, respectively (Table 1). Finally, *E. ceratoniae* displayed the highest sex ratio on the Shahvare-Danesefid cultivar, while the lowest one was seen on the Esfahani-Daneghermez and Malase Danesyah cultivars (Table 1).

**TABLE 1:** The mean (± SE) duration (days) and survival rate (%) of immature stages and sex ratio of *Ectomyelois ceratoniae* fed on various pomegranate cultivars.

**Pomegranate**	**Egg incubation**	**Larval period**	**Pre-pupal period**	**Pupal period**	**Development time**	**Pre-adult**	**Sex ratio**
**cultivar**	**(days)**	**(days)**	**(days)**	**(days)**	**(days)**	**survival**	
Aban-Mahi	3.77 ± 0.06 cd (52)	26.49 ± 0.32 ad (46)	1.98 ± 0.020 a (45)	6.31 ± 0.08 b (45)	38.58 ± 0.33 c (45)	0.76 ± 0.05 ab (60)	0.37 ± 0.06 ab (60)
Esfahani-Daneghermez	4.21 ± 0.06 a (51)	28.59 ± 0.24 a (45)	1.99 ± 0.002 a (43)	6.93 ± 0.04 a (41)	41.80 ± 0.26 a (41)	0.68 ± 0.06 b (60)	0.30 ± 0.06 b (60)
Gabri	3.96 ± 0.04 b (55)	27.92 ± 0.23 b (51)	1.96 ± 0.030 a (48)	6.19 ± 0.08 b (47)	39.88 ± 0.24 b (47)	0.78 ± 0.05 ab (60)	0.37 ± 0.06 ab (60)
Gorche-Tafti	3.84 ± 0.05 cd (55)	25.72 ± 0.21 de (52)	1.96 ± 0.030 a (51)	5.86 ± 0.07 cd (50)	37.43 ± 0.23 d (50)	0.83 ± 05 ab (60)	0.40 ± 0.07 ab (60)
Malase-Danesyah	4.20 ± 0.06 a (49)	28.31 ± 0.24 ab (46)	1.99 ± 0.005 a (44)	6.88 ± 0.05 a (42)	41.46 ± 0.28 a (42)	0.70 ± 0.06 b (60)	0.32 ± 0.06 b (60)
Malase-Yazdi	3.89 ± 0.06 bcd (53)	26.71 ± 0.25 c (48)	1.94 ± 0.030 ab (46)	6.29 ± 0.09 b (45)	38.62 ± 0.28 c (45)	0.75 ± 0.05 ab (60)	0.35 ± 0.06 ab (60)
Shahvare-Daneghermez	3.91 ± 0.04 bc (53)	26.77 ± 0.30 c (50)	1.99 ± 0.003 a (47)	6.25 ± 0.09 b (47)	39.03 ± 0.30 c (47)	0.79 ± 0.05 ab (60)	0.36 ± 0.06 ab (60)
Shahvare-Danesefid	3.80 ± 0.05 cd (57)	24.86 ± 0.24 f (54)	1.77 ± 0.060 b (53)	5.92 ± 0.09 c (52)	36.41 ± 0.31 f (52)	0.87 ± 0.05 a (60)	0.44 ± 0.07 a (60)
Tabolarze-Mehrmahi	3.83 ± 0.05 cd (54)	26.46 ± 0.25 cd (52)	1.99 ± 0.003 a (50)	6.17 ± 0.07 b (48)	38.48 ± 0.29 c (48)	0.80 ± 0.05 ab (60)	0.37 ± 0.06 ab (60)
Tafti	3.87 ± 0.04 bcd (56)	25.85 ± 0.23 d (51)	1.99 ± 0.002 a (50)	5.83 ± 0.07 cd (48)	37.67 ± 0.24 d (48)	0.81 ± 0.05 ab (60)	0.40 ± 0.06 ab (60)
Toghe-Gardan	3.73 ± 0.06 d (57)	25.09 ± 0.27 ef (52)	1.96 ± 0.030 a (51)	5.66 ± 0.07 d (50)	36.43 ± 0.28 e (50)	0.84 ± 0.05 ab (60)	0.42 ± 0.06 ab (60)

*Mean values in a column followed by different lowercase letters are significantly different (*P* < 0.05, paired bootstrap test). The numbers in parentheses indicate sample size.*

**FIGURE 1:**
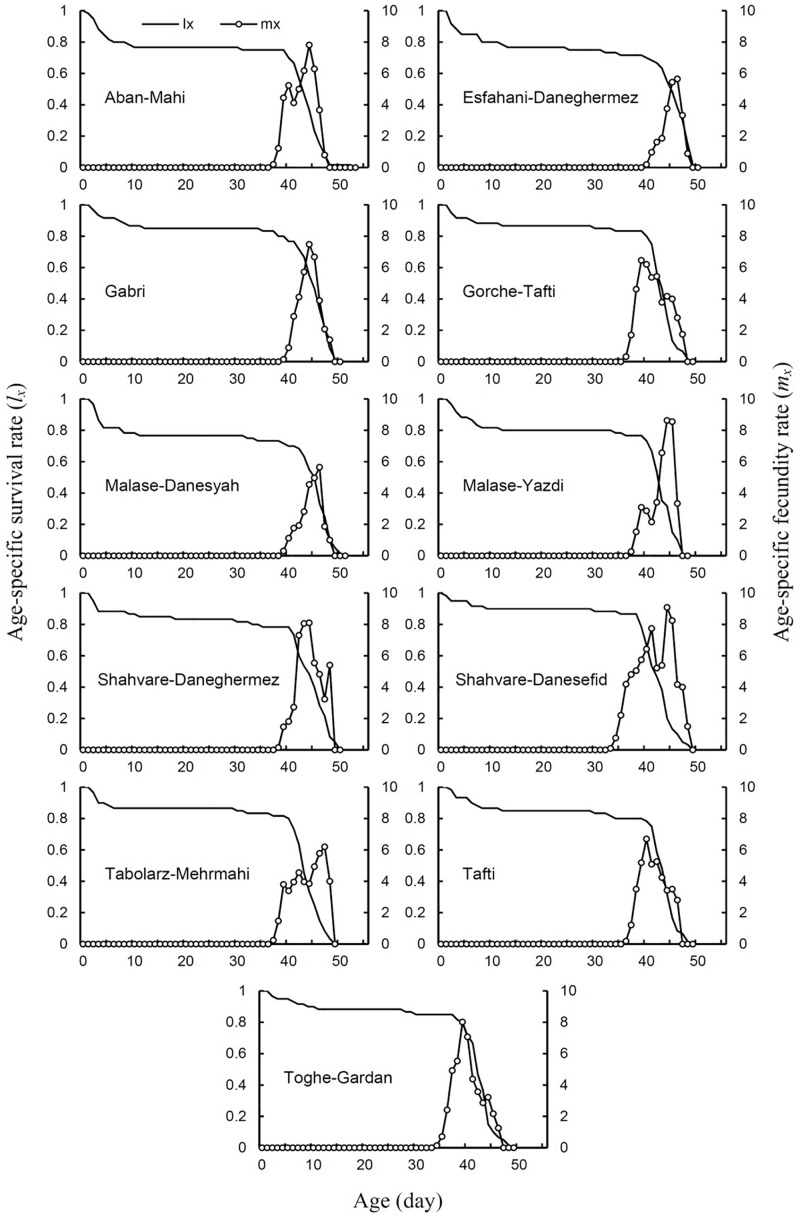
Age-specific survival rate (*l*_*x*_) and age-specific fecundity (*m*_*x*_) of *Ectomyelois ceratoniae* on various pomegranate cultivars.

The age-specific survival rate (*l*_*x*_) of *E. ceratoniae* on different cultivars is shown in Figure 1. Overall, age-specific survival rate curves were similar among insects fed different cultivars. However, female adults were longer-lived on the Aban-Mahi cultivar and the *l*_*x*_ curve was more extended on this cultivar.

Table 2 shows the weight of fifth instars and pupae of *E. ceratoniae* when reared on various pomegranate cultivars. The mean weight of fifth-instar larvae varied from 0.0425 ± 0.001 g on the Malase-Danesyah cultivar to 0.0615 ± 0.001 g on Shahvare-Danesefid (*F* = 31.66; df = 10, 546; *P* < 0.0001). Moreover, pupal weight showed significant variation as a function of the pomegranate cultivar tested (*F* = 25.35; df = 10, 514; *P* < 0.0001). The highest values were observed on the Shahvare-Danesefid and Toghe-Gardan cultivars and the lowest ones were recorded on the Malase-Danesyah and Esfahani-Daneghermez cultivars (Table 2).

**TABLE 2:** The mean (± SE) (g) weight of fifth instar larvae and pupa stage of *Ectomyelois ceratoniae* reared on various pomegranate cultivars.

**Pomegranate cultivar**	**Fifth instar larvae**	**Pupa**
Aban-Mahi	0.0504 ± 0.001 cd (46)	0.0410 ± 0.001 bc (45)
Esfahani-Danehghermez	0.0435 ± 0.001 ef (45)	0.0347 ± 0.001 e (41)
Gabri	0.0468 ± 0.001 def (51)	0.0387 ± 0.001 cd (47)
Gorche-Tafti	0.0542 ± 0.001 bc (52)	0.0418 ± 0.001 abc (50)
Malase-Danehsyah	0.0425 ± 0.001 f (46)	0.0342 ± 0.001 e (42)
Malase-Yazdi	0.0466 ± 0.001 def (48)	0.0362 ± 0.001 de (45)
Shahvare-Danehghermez	0.0455 ± 0.001 def (50)	0.0391 ± 0.001 bcd (47)
Shahvare-Danehsefid	0.0615 ± 0.001 a (54)	0.0442 ± 0.001 a (52)
Tabolarze-Mehrmahi	0.0484 ± 0.001 de (52)	0.0393 ± 0.001 bcd (48)
Tafti	0.0566 ± 0.001 b (51)	0.0420 ± 0.001 ab (48)
Toghe-Gardan	0.0581 ± 0.002 ab (52)	0.0448 ± 0.001 a (50)

*Mean values in a column followed by different lowercase letters are significantly different on the basis of ANOVA with Tukey test (*P* < 0.05). The numbers in parentheses indicate sample size.*

### Longevity and Reproductive Variables

The data on pre-oviposition and oviposition periods, fecundity and lifespan of *E. ceratoniae* adults on the tested pomegranate cultivars are presented in Table 3. Significant effects were observed for adult pre-oviposition period (APOP) of *E. ceratoniae*. Total pre-oviposition period (TPOP) varied significantly as a function of the pomegranate cultivars used as food source. The TPOP was longest on the Esfahani-Daneghermez cultivar and shortest on Toghe-Gardan. Moreover, the oviposition period was the longest when *E. ceratoniae* females were reared on the Shahvare-Danesefid cultivar and the shortest when reared on the Esfahani-Daneghermez, Malase-Danesyah, and Malase-Yazdi cultivars. Total fecundity (mean number of eggs laid during the reproductive period) of *E. ceratoniae* varied significantly as a function of the 11 pomegranate cultivars tested; the highest values were observed on the Shahvare-Danesefid (82.74 ± 2.01 eggs) and Toghe-Gardan (70.46 ± 2.30 eggs) cultivars and the lowest was on Esfahani-Daneghermez (36.91 ± 1.35 eggs) (Table 3).

**TABLE 3:** Biological parameters of *Ectomyelois ceratoniae* adults reared on various pomegranate cultivars.

**Pomegranate**	**APOP**	**TPOP**	**Oviposition period**	**Fecundity**	**Female adult**	**Male adult**
**cultivar**	**(days)**	**(days)**	**(days)**	**(no. eggs laid)**	**longevity (days)**	**longevity (days)**
Aban-Mahi	1.04 ± 0.05 b (22)	39.92 ± 0.41 ef (22)	4.55 ± 0.23 cd (22)	58.61 ± 2.05 c (22)	6.27 ± 0.25 bc (22)	5.47 ± 0.20 c (23)
Esfahani-Daneghermez	1.22 ± 0.10 ab (18)	43.11 ± 0.40 a (18)	3.39 ± 0.18 e (18)	36.91 ± 1.35 f (18)	5.33 ± 0.19 d (18)	4.47 ± 0.23 d (23)
Gabri	1.18 ± 0.08 ab (22)	41.64 ± 0.28 bc (22)	4.22 ± 0.18 d (22)	51.51 ± 1.94 d (22)	6.14 ± 0.23 c (22)	5.76 ± 0.23 abc (25)
Gorche-Tafti	1.17 ± 0.07 ab (24)	38.52 ± 0.32 gh (24)	5.03 ± 0.25 abc (24)	67.03 ± 2.28 b (24)	6.82 ± 0.27 abc (24)	5.96 ± 0.14 ab (26)
Malase-Danesyah	1.16 ± 0.09 ab (19)	42.23 ± 0.45 ab (19)	3.68 ± 0.19 e (19)	40.37 ± 1.63 ef (19)	5.53 ± 0.16 d (19)	4.57 ± 0.22 d (23)
Malase-Yazdi	1.28 ± 0.10 a (21)	40.30 ± 0.49 de (21)	3.67 ± 0.20 e (21)	44.97 ± 2.02 e (21)	5.48 ± 0.23 d (21)	4.91 ± 0.21 d (24)
Shahvare-Daneghermez	1.18 ± 0.08 ab (22)	41.09 ± 0.33 cd (22)	5.01 ± 0.17 abc (22)	60.02 ± 1.99 c (22)	6.90 ± 0.23 ab (22)	5.72 ± 0.15 abc (25)
Shahvare-Danesefid	1.11 ± 0.06 ab (26)	37.45 ± 0.52 hi (26)	5.50 ± 0.20 a (26)	82.74 ± 2.01 a (26)	7.04 ± 0.21 a (26)	5.84 ± 0.18 abc (26)
Tabolarze-Mehrmahi	1.09 ± 0.06 ab (22)	39.72 ± 0.43 ef (22)	4.64 ± 0.23 bcd (22)	51.33 ± 2.08 d (22)	6.28 ± 0.23 bc (22)	5.51 ± 0.20 bc (26)
Tafti	1.21 ± 0.09 ab (24)	38.92 ± 0.37 fg (24)	4.66 ± 0.23 bcd (24)	60.34 ± 2.29 c (24)	6.82 ± 0.27 abc (24)	5.96 ± 0.15 ab (24)
Toghe-Gardan	1.08 ± 0.05 ab (25)	37.23 ± 0.35 i (25)	5.16 ± 0.20 ab (25)	70.46 ± 2.30 a (25)	7.19 ± 0.27 a (25)	6.04 ± 0.16 a (25)

*Mean values in a column followed by different lowercase letters are significantly different (*P* < 0.05, paired bootstrap test). The numbers in parentheses indicate sample size. APOP, adult pre-oviposition period; TPOP, total pre-oviposition period.*

Age-specific fecundity rates (*m*_*x*_) for *E. ceratoniae* reared on the pomegranate cultivars tested are shown in Figure 1. The width of the *m*_*x*_ curve (i.e., the fecundity period) was the greater on the Shahvare-Danesefid cultivar than the others.

Longevity of both male and female adults of *E. ceratoniae* was lowest when larvae were reared on the Esfahani-Daneghermez, Malase-Danesyah, and Malase-Yazdi cultivars (Table 3).

### Adult Life Table Parameters

Life tables were constructed from developmental time, survival, and fecundity data (Table 4). All computed parameters were significantly affected by the pomegranate cultivar. Insects reared on Shahvare-Danesefid had the highest gross reproductive rate (*GRR*) while those reared on Esfahani-Daneghermez showed the lowest *GRR* value. The net reproductive rate (*R*_0_) was the lowest on the Esfahani-Daneghermez cultivar and the highest on the Shahvare-Danesefid cultivar. The intrinsic rate of increase (*r*_*m*_) values ranged from 0.053 ± 0.004 to 0.090 ± 0.004 female progeny per female per day on the pomegranate cultivars under study (Table 4). The *r*_*m*_ value was highest when *E. ceratoniae* were reared on the Shahvare-Danesefid cultivar and lowest on the Esfahani-Daneghermez cultivar. Variation in the finite rate of increase (λ) was similar to that observed for the intrinsic rate of increase while the mean generation time (*T*) of *E. ceratoniae* was shortest on the Toghe-Gardan cultivar (Table 4).

**TABLE 4:** Mean (± SE) of two-sex life-table parameters for *Ectomyelois ceratoniae* reared on various pomegranate cultivars.

**Pomegranate cultivar**	**Sample**	***GRR***	***R*_0_**	***r*_*m*_**	**λ**	***T* (days)**
	**size (n)**	**(female/female)**	**(female/female)**	**(female/female/day)**	**(female/day)**	
Aban-Mahi	60	44.98 ± 6.71 b	21.59 ± 3.65 bc	0.072 ± 0.004 cde	1.075 ± 0.004 cde	42.50 ± 0.48 c
Esfahani-Daneghermez	60	23.90 ± 4.30 c	5.27 ± 4.46 e	0.053 ± 0.004 g	1.054 ± 0.005 g	45.33 ± 0.38 a
Gabri	60	35.44 ± 5.51 bc	19.05 ± 3.30 bcd	0.066 ± 0.004 def	1.068 ± 0.004 def	44.26 ± 0.30 b
Gorche-Tafti	60	46.48 ± ±7.40 b	26.83 ± 4.49 ab	0.080 ± 0.004 abc	1.083 ± 0.005 abc	41.10 ± 0.33 fg
Malase-Danesyah	60	25.85 ± 4.64 c	12.71 ± 2.47 d	0.057 ± 0.005 fg	1.058 ± 0.005 fg	44.56 ± 0.40 ab
Malase-Yazdi	60	40.50 ± 5.53 b	15.67 ± 2.84 cd	0.064 ± 0.004 efg	1.066 ± 0.005 efg	42.44 ± 0.41 cd
Shahvare-Daneghermez	60	48.47 ± 7.12 b	21.84 ± 3.87 bc	0.070 ± 0.004 cde	1.073 ± 0.004 cde	43.84 ± 0.36 b
Shahvare-Danesefid	60	74.64 ± 10.55 a	36.05 ± 5.60 a	0.090 ± 0.004 a	1.093 ± 0.004 a	40.02 ± 0.50 gh
Tabolarze-Mehrmahi	60	45.40 ± 8.44 b	18.88 ± 3.31 bcd	0.069 ± 0.004 cde	1.071 ± 0.005 cde	42.34 ± 0.44 cde
Tafti	60	41.03 ± 5.85 b	24.01 ± 3.84 abc	0.077 ± ±0.004 bcd	1.080 ± 0.004 bcd	41.34 ± 0.34 ef
Toghe-Gardan	60	46.29 ± 7.84 b	29.29 ± 4.58 ab	0.084 ± 0.004 ab	1.088 ± 0.004 ab	39.90 ± 0.32 h

*Mean values in a column followed by different lowercase letters are significantly different (*P* < 0.05, paired bootstrap test). *GRR*, gross reproductive rate; *R*_0_, net reproductive rate; *r*_*m*_, intrinsic rate of increase; λ, finite rate of increase; *T*, mean generation time.*

### Nutritional Indices and Storage Macromolecules of Larvae

Measurements of the nutritional performance of *E. ceratoniae* fourth-instar larvae are shown in Table 5. Nutritional indices were significantly different among larvae feeding on various pomegranate cultivars. The larvae fed on Shahvare-Danesefid showed a higher level of food consumption as compared with those reared on other cultivars (*F* = 23.15; df = 10, 439; *P* < 0.0001). Also, the highest weight gain of larvae (*F* = 47.91; df = 10, 439; *P* < 0.0001) was observed when larvae were fed on Shahvare-Danesefid (Table 5). Larvae reared on the Shahvare-Danesefid and Gorche-Tafti cultivars showed the highest efficiency of conversion of ingested food (ECI) (*F* = 22.89; df = 10, 439; *P* < 0.0001) as well as efficiency of conversion of digested food (ECD) (*F* = 22.77; df = 10, 439; *P* < 0.0001) compared with larvae fed on the other cultivars (Table 5). The highest RCR (*F* = 2.48; df = 10, 439; *P* = 0.0068) was observed on the Esfahani-Daneghermez and Tabolarze-MehrMahi cultivars, while larvae reared on Gorche-Tafti had a lowest RCR. In addition, the highest value of relative growth rate (RGR) (*F* = 7.93; df = 10, 439; *P* < 0.0001) was obtained for larvae reared on the Shahvare-Danesefid cultivar (Table 5).

**TABLE 5:** The mean (± SE) nutritional indices of *Ectomyelois ceratoniae* reared on various pomegranate cultivars.

**Pomegranate cultivar**	**Sample**	**Food consumed**	**Larval weight**	**ECI (%)**	**ECD (%)**	**RCR (g/g/day)**	**RGR (g/g/day)**
	**size (n)**	**(g/larvae)**	**gain (g)**				
Aban-Mahi	40	2.70 ± 0.06 cd	0.020 ± 0.002 de	1.93 ± ±0.12 bc	1.99 ± ±0.13 bc	4.11 ± ±0.14 ab	0.076 ± ±0.004 bc
Esfahani-Danehghermez	40	2.59 ± ±0.04 d	0.014 ± 0.001 e	1.44 ± ±0.08 d	1.49 ± ±0.08 d	4.53 ± ±0.14 a	0.064 ± ±0.004 c
Gabri	40	2.65 ± ±0.05 cd	0.016 ± 0.001 e	1.57 ± ±0.09 cd	1.62 ± ±0.09 cd	4.24 ± ±0.14 ab	0.065 ± ±0.004 c
Gorche-Tafti	40	3.10 ± 0.07 b	0.031 ± ±0.008 b	2.69 ± ±0.10 a	2.78 ± ±0.10 a	3.41 ± ±0.16 b	0.090 ± ±0.005 ab
Malase-Danehsyah	40	2.63 ± ±0.05 cd	0.015 ± ±0.009 e	1.47 ± ±0.08 d	1.52 ± ±0.08 d	4.36 ± ±0.14 ab	0.063 ± ±0.004 c
Malase-Yazdi	40	2.62 ± 0.05 cd	0.016 ± 0.004 e	1.59 ± ±0.10 cd	1.64 ± ±0.10 cd	4.25 ± ±0.14 ab	0.065 ± ±0.004 c
Shahvare-Danehghermez	40	2.66 ± ±0.04 cd	0.017 ± 0.003 e	1.69 ± ±0.11 bcd	1.74 ± ±0.11 bcd	4.20 ± ±0.16 ab	0.067 ± ±0.004 c
Shahvare-Danehsefid	40	3.62 ± 0.07 a	0.039 ± ±0.006 a	2.84 ± ±0.08 a	2.92 ± ±0.08 a	3.74 ± ±0.12 ab	0.105 ± ±0.003 a
Tabolarze-Mehrmahi	40	2.76 ± 0.07 cd	0.019 ± 0.001 de	1.78 ± ±0.12 bcd	1.84 ± ±0.13 bcd	4.63 ± ±0.64 a	0.074 ± ±0.007 bc
Tafti	40	2.80 ± ±0.09 cd	0.023 ± 0.001 cd	2.09 ± ±0.10 b	2.18 ± ±0.11 b	3.91 ± ±0.17 ab	0.080 ± ±0.005 bc
Toghe-Gardan	40	2.89 ± 0.08 bc	0.025 ± ±0.003 c	2.14 ± ±0.09 b	2.20 ± ±0.10 b	3.66 ± ±0.18 ab	0.076 ± ±0.004 bc

*The means followed by different letters in the same column are significantly different (Tukey test, *P* < 0.05). ECI, efficiency of conversion of ingested food; ECD, efficiency of conversion of digested food; RCR, relative consumption rate; RGR, relative growth rate.*

Values measured for the energy reserves of *E. ceratoniae* fourth-instar larvae on various pomegranate cultivars are given in Table 6. There was significant variation in energy contents of *E. ceratoniae* larvae reared on different cultivars. The highest whole-body protein content of larvae (*F* = 16.05; df = 10, 32; *P* = 0.0068) was observed in those fed on the Shahvare-Danesefid cultivar (*F* = 3.16; df = 10, 32; *P* = 0.0118). Similarly, the larvae fed on this cultivar showed the highest whole-body glycogen content (*F* = 3.16; df = 10, 32; *P* = 0.0118). Larvae fed on the Shahvare-Danesefid and Tafti cultivars showed the highest lipid content (*F* = 11.07; df = 10, 32; *P* < 0.0001) while the lowest value was observed for larvae reared on the Malase-Danesyah cultivar (Table 6).

**TABLE 6:** The mean (± SE) energy reserves of fourth-instar larvae of *Ectomyelois ceratoniae* reared on various pomegranate cultivars.

**Pomegranate cultivar**	**Sample size (n)**	**Protein content (μg/larva)**	**Glycogen content (μg/larva)**	**Lipid content (μg/larva)**
Aban-Mahi	3	491.67 ± 8.90 cde	58.45 ± 6.27 ab	59.87 ± 7.59 abc
Esfahani-Danehghermez	3	468.67 ± 1.20 e	39.63 ± 12.16 ab	45.89 ± 4.59 cd
Gabri	3	492.02 ± 5.57 cde	40.33 ± 11.19 ab	52.85 ± 3.56 cd
Gorche-Tafti	3	505.50 ± 8.13 bcd	63.29 ± 6.87 ab	67.12 ± 7.83 abc
Malase-Danehsyah	3	482.33 ± 6.86 de	29.90 ± 8.41 b	34.15 ± 6.15 d
Malase-Yazdi	3	494.67 ± 7.66 bcde	38.45 ± 3.73 ab	47.05 ± 3.49 cd
Shahvare-Danehghermez	3	503.33 ± 9.70 bcde	50.60 ± 4.45 ab	60.17 ± 3.75 abc
Shahvare-Danehsefid	3	574.33 ± 7.22 a	73.34 ± 10.71 a	81.83 ± 1.66 a
Tabolarze-Mehrmahi	3	502.50 ± 4.77 bcde	46.95 ± 2.82 ab	56.40 ± 2.39 bcd
Tafti	3	520.01 ± 5.69 bc	65.60 ± 2.84 ab	80.02 ± 1.90 a
Toghe-Gardan	3	529.83 ± 3.01 b	70.60 ± 12.31 ab	77.20 ± 1.26 ab

*Mean values in a column followed by different lowercase letters are significantly different on the basis of ANOVA with Tukey test (*P* < 0.05).*

**FIGURE 2:**
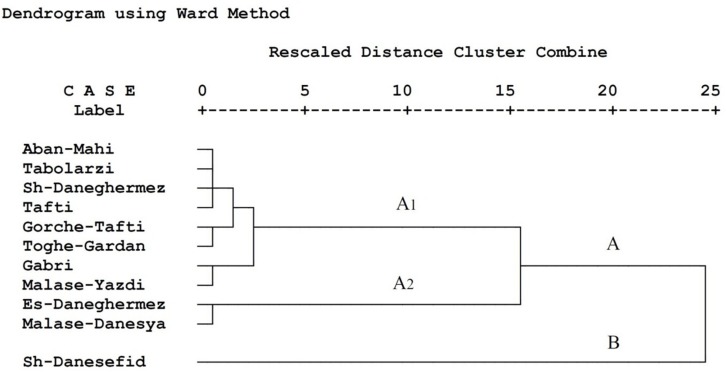
Dendrogram of different pomegranate cultivars based on life table parameters, nutritional indices and energy reserves of *Ectomyelois ceratoniae* reared on various pomegranate cultivars.

A dendrogram based on population growth parameters, nutritional indices, and energy reserves of *E. ceratoniae* on various pomegranate cultivars is shown in Figure 2. The dendrogram revealed two clusters labeled A (including sub-clusters A1 and A2) and B. Different pomegranate cultivars tested were grouped within each cluster according to the *E. ceratoniae* criteria. Cluster B included Shahvare-Danesefid; sub-cluster A2 consisted of Esfahani-Daneghermez and Malase-Danesyah and other cultivars were in sub-cluster A1.

### Biochemical Traits of Pomegranate Cultivars

Concentrations of the major anthocyanins measured in the pomegranate cultivars that we examined are listed in Table 7. Six compounds, namely cyanidin 3-glucoside (0.48–11.91 mg/L), cyanidin 3,5-diglucoside (1.90–54.47 mg/L), delphinidin 3-glucoside (0.78–4.28 mg/L), delphinidin 3,5-diglucoside (0.072–40.59 mg/L), pelargonidin 3-glucoside (0.118–0.55 mg/L), and pelargonidin 3,5-diglucoside (0.37–3.52 mg/L), were detected as major anthocyanin constituents from various pomegranate juice cultivars. There were significant differences among various pomegranate cultivars for all the detected anthocyanin compounds (Table 7). Esfahani-Daneghermez cultivar showed the highest value of cyanidin 3-glucoside (*F* = 710.64; df = 10, 32; *P* < 0.0001) compound compared with other cultivars. The cyanidin 3,5-diglucoside (*F* = 979.90; df = 10, 32; *P* < 0.0001) was significantly higher in Esfahani-Daneghermez. Also, the highest value of delphinidin 3-glucoside (*F* = 473.38; df = 10, 32; *P* < 0.0001), delphinidin 3,5-diglucoside (*F* = 24.31; df = 10, 32; *P* < 0.0001), pelargonidin 3-glucoside (*F* = 52.35; df = 10, 32; *P* < 0.0001) and pelargonidin 3,5-diglucoside (*F* = 12.74; df = 10, 32; *P* < 0.0001) compounds were measured in Esfahani-Daneghermez (Table 7).

**TABLE 7:** The means (± SE) major anthocyanins constituents (mg/L) of various pomegranate cultivars.

**Pomegranate**	**Sample**	**Cyanidin**	**Cyanidin**	**Delphinidin**	**Delphinidin**	**Pelargonidin**	**Pelargonidin**
**cultivar**	**size (n)**	**3-glucoside**	**3,5-diglucoside**	**3-glucoside**	**3,5 diglucoside**	**3- glucoside**	**3,5- diglucoside**
Aban-Mahi	3	2.13 ± 0.05d	7.73 ± 0.37d	1.19 ± 0.02e	1.38 ± 0.40c	0.17 ± 0.010bcd	0.56 ± 0.040d
Esfahani-Danehghermez	3	11.91 ± 0.04a	54.47 ± 1.47a	4.28 ± 0.09a	40.59 ± 7.94a	0.51 ± 0.020a	3.52 ± 0.080a
Gabri	3	2.61 ± 0.03d	13.36 ± 0.24c	1.56 ± 0.02d	2.61 ± 0.07bc	0.24 ± 0.006bc	1.17 ± 0.020bcd
Gorche-Tafti	3	1.11 ± 0.02e	4.17 ± 0.03ef	0.95 ± 0.00ef	1.28 ± 0.03c	0.13 ± 0.001*cd*	0.43 ± 0.020d
Malase-Danehsyah	3	5.44 ± 0.12c	39.83 ± 0.96b	3.52 ± 0.15b	2.01 ± 0.57b	0.25 ± 0.004b	2.36 ± 0.090ab
Malase-Yazdi	3	9.94 ± 0.24b	37.44 ± 0.64b	3.16 ± 0.04c	9.33 ± 0.70b	0.55 ± 0.070a	2.92 ± 0.280a
Shahvare-Danehghermez	3	0.48 ± 0.06e	6.02 ± 0.17de	1.06 ± 0.03ef	1.42 ± 0.01c	0.15 ± 0.002bcd	1.14 ± 0.010bcd
Shahvare-Danehsefid	3	0.86 ± 0.01e	3.39 ± 0.04ef	0.90 ± 0.01f	0.072 ± 0.01c	0.118 ± 0.001d	0.37 ± 0.002d
Tabolarze-Mehrmahi	3	1.16 ± 0.38e	15.81 ± 0.02c	1.72 ± 0.00d	0.197 ± 0.05c	0.16 ± 0.006bcd	2.23 ± 0.910abc
Tafti	3	0.64 ± 0.13e	1.90 ± 0.07f	0.78 ± 0.02f	0.81 ± 0.06c	0.119 ± 0.001d	0.74 ± 0.390cd
Toghe-Gardan	3	0.83 ± 0.01e	3.61 ± 0.13ef	0.92 ± 0.01ef	0.89 ± 0.04c	0.122 ± 0.002d	0.37 ± 0.010d

*Mean values in a column followed by different lowercase letters are significantly different on the basis of ANOVA with Tukey test (*P* < 0.05).*

Biochemical characteristics of tested pomegranate cultivars used for feeding of *E. ceratoniae* are given in Tables 8, 9. No significant differences were observed in total protein content (*F* = 1.10; df = 10, 32; *P* = 0.41) among the pomegranate cultivars. The carbohydrate content varied from 92.61 mg/mL in Esfahani-Daneghermez to 138.33 mg/mL in Shahvare-Danesefid cultivar (*F* = 16.22; df = 10, 32; *P* < 0.0001). Furthermore, a higher content of condensed tannins was measured in Esfahani-Daneghermez and Shahvare-Daneghermez, whereas a lower content was detected in Shahvare-Danesefid and Toghe-Gardan cultivars (*F* = 12.18; df = 10, 32; *P* < 0.0001) (Table 8). There was a significant difference for the total phenolic (TPC) (*F* = 45.22; df = 10, 32; *P* < 0.0001) and total monomeric anthocyanin pigment (TMAC) (*F* = 38.99; df = 10, 32; *P* < 0.0001) contents of different pomegranate cultivars. A higher value of TPC and TMAC was measured in Esfahani-Daneghermez cultivar and the lowest value of TMAC was detected in Shahvare-Danesefid and Tafti cultivars. The highest flavonoid content was measured in Esfahani-Daneghermez, whereas the lowest content was observed in the Shahvare-Danesefid and Toghe-Gardan cultivars (*F* = 44.70; df = 10, 32; *P* < 0.0001) (Table 8).

**TABLE 8:** Some biochemical characteristics of tested various pomegranate juice cultivars.

			**Carbohydrate**				
**Pomegranate**	**Sample**	**Total Protein**	**content**	**CT**	**TPC**	**TMAC**	**Flavonoid**
**cultivar**	**size (n)**	**(mg/mL)**	**(mg/mL)**	**(mg/mL)**	**(mg/100 mL)**	**(mg/L)**	**(mg/mL)**
Aban-Mahi	3	0.139 ± 0.004a	102.78 ± 4.99bc	20.01 ± 1.05abcd	277.35 ± 9.33abc	138.07 ± 8.09cde	98.37 ± 6.15b
Esfahani-Danehghermez	3	0.133 ± 0.003a	92.61 ± 5.61c	23.55 ± 0.58a	308.61 ± 1.21a	244.52 ± 5.81a	132.44 ± 11.37a
Gabri	3	0.132 ± 0.002a	97.06 ± 3.19bc	21.08 ± 1.14abc	246.68 ± 11.13bcd	152.24 ± 8.50bcd	90.84 ± 0.83b
Gorche-Tafti	3	0.136 ± 0.005a	131.45 ± 0.89a	16.21 ± 1.39cde	165.35 ± 6.96ef	99.69 ± 13.72efg	40.44 ± 3.46cd
Malase-Danehsyah	3	0.130 ± 0.002a	94.39 ± 4.36bc	21.55 ± 0.57ab	294.68 ± 5.77ab	172.93 ± 8.46bc	108.04 ± 4.45ab
Malase-Yazdi	3	0.136 ± 0.002a	95.44 ± 6.65bc	19.95 ± 0.74abcd	259.68 ± 1.73abc	181.20 ± 4.69b	98.77 ± 3.48b
Shahvare-Danehghermez	3	0.134 ± 0.001a	117.67 ± 0.92ab	23.15 ± 0.71a	202.68 ± 25.16de	169.73 ± 5.37bcd	63.11 ± 6.36c
Shahvare-Danehsefid	3	0.138 ± 0.002a	138.33 ± 0.73a	14.01 ± 0.64e	131.35 ± 4.37f	90.78 ± 7.92*g*	27.11 ± 3.53d
Tabolarze-Mehrmahi	3	0.131 ± 0.002a	99.61 ± 5.24bc	18.01 ± 1.80bcde	236.68 ± 9.02cd	132.31 ± 3.09def	93.11 ± 3.71b
Tafti	3	0.134 ± 0.001a	134.61 ± 1.02a	16.01 ± 0.70de	144.68 ± 5.29f	91.38 ± 8.23*g*	37.77 ± 4.66cd
Toghe-Gardan	3	0.133 ± 0.004a	135.83 ± 1.11a	14.28 ± 0.46e	130.68 ± 3.46f	95.83 ± 5.37fg	32.44 ± 4.16d

*Mean values in a column followed by different lowercase letters are significantly different on the basis of ANOVA with Tukey test (*P* < 0.05). CT, condensed tannins; TPC, total phenolic content; TMAC, total monomeric anthocyanin pigment content.*

**TABLE 9:** Some biochemical characteristics of tested various pomegranate juice cultivars.

**Pomegranate cultivar**	**Sample size (n)**	**DPPH (inhibition%)**	**pH**	**TA (g/L)**	**Juice (%)**	**SSC (°Brix)**
Aban-Mahi	3	51.71 ± 0.58cd	3.55 ± 0.08de	2.33 ± 0.07bc	51.13 ± 1.65b	16.67 ± 0.67ab
Esfahani-Danehghermez	3	60.13 ± 0.96a	3.30 ± 0.08ef	2.73 ± 0.07abc	53.42 ± 3.55b	17.01 ± 0.58a
Gabri	3	57.80 ± 2.39abc	3.38 ± 0.05ef	2.79 ± 0.23ab	57.04 ± 2.17ab	17.17 ± 0.44a
Gorche-Tafti	3	50.83 ± 0.57d	4.07 ± 0.09ab	1.33 ± 0.13ef	61.18 ± 1.08a	15.33 ± 0.34bc
Malase-Danehsyah	3	58.48 ± 2.54ab	3.21 ± 0.05f	2.99 ± 0.11a	51.68 ± 2.39b	17.33 ± 0.67a
Malase-Yazdi	3	54.47 ± 0.51abcd	3.28 ± 0.04ef	2.87 ± 0.18ab	56.50 ± 3.29ab	16.83 ± 0.44ab
Shahvare-Danehghermez	3	52.23 ± 1.13bcd	3.73 ± 0.08cd	2.13 ± 0.13cd	55.80 ± 1.38ab	16.33 ± 0.33abc
Shahvare-Danehsefid	3	50.94 ± 0.95d	4.15 ± 0.04a	0.93 ± 0.13f	61.32 ± 1.02a	16.01 ± 0.58abc
Tabolarze-Mehrmahi	3	54.48 ± 0.51abcd	3.83 ± 0.05bcd	1.60 ± 0.11de	50.15 ± 1.78b	15.01 ± 0.58c
Tafti	3	49.17 ± 1.20d	4.01 ± 0.07abc	1.27 ± 0.06ef	54.82 ± 1.11ab	17.33 ± 0.67a
Toghe-Gardan	3	49.94 ± 0.67d	3.96 ± 0.04abc	1.40 ± 0.11ef	52.38 ± 3.77b	17.5 ± 0.51a

*Mean values in a column followed by different lowercase letters are significantly different on the basis of ANOVA with Tukey test (*P* < 0.05). DPPH, 2,2 diphenyl-1-picrylhydrazyl; TA, total titrable acidity; SSC, soluble solids content.*

Antioxidant activity (AA%) varied from 49.17 in Tafti to 60.13 (AA%) in the Esfahani-Daneghermez cultivar (*F* = 8.30; df = 10, 32; *P* < 0.0001). Moreover, the highest pH value was observed in the Shahvare-Danesefid cultivar while the lowest value was in the Malase-Danesyah cultivar (*F* = 34.32; df = 10, 32; *P* < 0.0001). The highest total titrable acidity value was obtained for the Malase-Danesyah cultivar, whereas the lowest value was seen for the Shahvare-Danesefid cultivar (*F* = 24.37; df = 10, 32; *P* < 0.0001). The percentage of juice showed limited variation, ranging from 50.15 to 61.32 for the pomegranate cultivars under investigation (*F* = 2.66; df = 10, 32; *P* = 0.0265). The soluble solids content was lowest in the Tabolarze-Mehrmahi cultivar (*F* = 2.42; df = 10, 32; *P* = 0.040) (Table 9).

### Correlation Analysis

Tables 10, 11 show coefficients obtained for analyses of correlation between life table parameters and physiological characteristics of *E. ceratoniae* fed on different cultivars, on the one hand, and different biochemical traits of the various pomegranate cultivars tested, on the other hand; significant positive or negative correlations were observed (Tables 10, 11).

**TABLE 10:** Correlation coefficients (r) of some life history parameters and physiological characteristics of *Ectomyelois ceratoniae* fed on different pomegranate cultivars with major anthocyanins constituents of various pomegranate cultivars.

	**Cyanidin**	**Cyanidin**	**Delphinidin**	**Delphinidin**	**Pelargonidin**	**Pelargonidin**
**Parameter**	**3-glucoside**	**3,5-diglucoside**	**3-glucoside**	**3,5-diglucoside**	**3- glucoside**	**3,5-diglucoside**
Development time	0.665 (0.026)	0.813 (0.002)	0.819 (0.002)	0.606 (0.048)	0.593 (0.05)	0.758 (0.007)
Fecundity	−0.750 (0.008)	−0.850 (0.001)	−0.850 (0.001)	−0.571 (0.067)	−0.732 (0.011)	−0.877 (0.000)
Weight of pupa	−0.774 (0.005)	−0.890 (0.000)	−0.894 (0.000)	−0.555 (0.077)	−0.751 (0.008)	−0.888 (0.000)
Protein content of larva	−0.590 (0.056)	−0.664 (0.026)	−0.662 (0.026)	−0.503 (0.115)	−0.572 (0.066)	−0.660 (0.027)
*R*_0_	−0.790 (0.004)	−0.876 (0.000)	−0.872 (0.000)	−0.697 (0.017)	−0.752 (0.008)	−0.886 (0.000)
*r*_*m*_	−0.752 (0.008)	−0.866 (0.001)	−0.867 (0.001)	−0.619 (0.042)	−0.718 (0.013)	−0.867 (0.001)
ECI	−0.580 (0.062)	−0.683 (0.021)	−0.683 (0.021)	−0.424 (0.194)	−0.605 (0.049)	−0.742 (0.009)
RCR	0.510 (0.109)	0.659 (0.027)	0.655 (0.029)	0.420 (0.198)	0.523 (0.099)	0.796 (0.003)
RGR	−0.534 (0.090)	−0.619 (0.042)	−0.620 (0.042)	−0.371 (0.261)	−0.569 (0.068)	−0.664 (0.026)

*Correlations were evaluated based on Pearson’s correlation test (*P* < 0.05). The number in parenthesis is *P*_*value*_.*

**TABLE 11:** Correlation coefficients (r) of some life history parameters and physiological characteristics of *Ectomyelois ceratoniae* fed on different pomegranate cultivars with biochemical traits of various pomegranate cultivars.

**Parameter**	**Total pProtein**	**Carbohydrate**	**CT**	**TPC**	**TMAC**	**DPPH**
Development time	−0.517 (0.103)	−0.832 (0.001)	0.886 (0.000)	0.883 (0.000)	0.876 (0.000)	0.912 (0.000)
Fecundity	0.543 (0.084)	0.893 (0.000)	−0.794 (0.004)	−0.884 (0.000)	−0.841 (0.001)	−0.824 (0.002)
Weight of pupa	0.467 (0.147)	0.873 (0.000)	−0.846 (0.001)	−0.883 (0.000)	−0.888 (0.000)	−0.870 (0.001)
Protein content of larva	0.409 (0.212)	0.818 (0.002)	−0.821 (0.002)	−0.853 (0.001)	−0.770 (0.006)	−0.695 (0.018)
*R*_0_	0.490 (0.126)	0.867 (0.001)	−0.832 (0.001)	−0.890 (0.000)	−0.903 (0.000)	−0.839 (0.001)
*r*_*m*_	0.511 (0.108)	0.899 (0.000)	−0.881 (0.000)	−0.916 (0.000)	−0.908 (0.000)	−0.880 (0.000)
ECI	0.580 (0.061)	0.849 (0.001)	−0.834 (0.001)	−0.801 (0.003)	−0.803 (0.003)	−0.739 (0.009)
RCR	−0.504 (0.114)	−0.856 (0.001)	0.727 (0.011)	0.792 (0.004)	0.743 (0.009)	0.743 (0.009)
RGR	0.590 (0.056)	0.779 (0.005)	−0.802 (0.003)	−0.732 (0.010)	−0.751 (0.008)	−0.662 (0.026)

*Correlations were evaluated based on Pearson’s correlation test (*P* < 0.05). The number in parenthesis is *P*_*value*_.*

These analyses revealed high positive correlation between developmental time and contents of all major anthocyanin compounds of pomegranate cultivars, while a significant negative correlation was observed between *R*_0_ and *r*_*m*_, and all major anthocyanin contents (Table 10).

Fecundity and pupal weight, as well as the nutritional indices ECI and RGR, were also negatively correlated with contents of all major anthocyanins, whereas RCR was positively correlated with these same variables (Table 10).

The life history variables and physiological characteristics of *E. ceratoniae* listed in Table 11 were not significantly correlated with total protein contents of the various pomegranate cultivars. However, significant positive or negative correlations were observed for life history variables and physiological characteristics with carbohydrate and condensed tannins of pomegranate cultivars. Very high positive correlations were observed between the carbohydrate content of the cultivars and *E. ceratoniae* fecundity, pupal weight, protein content of larvae, *R*_0_ and *r*_*m*_; developmental time, on the other hand, was negatively correlated with this biochemical trait of pomegranate cultivars. Moreover, a significant negative correlation was observed between condensed tannins of various pomegranate cultivars and *E. ceratoniae* fecundity, pupal weight, protein content of larva, *R*_0_, *r*_*m*_, ECI and RGR (Table 11).

Significant correlations were observed for life history variables and physiological characteristics of *E. ceratoniae* with total phenolic content, total monomeric anthocyanin pigment content, and DPPH of pomegranate cultivars. Specifically, fecundity, pupal weight, protein content of larva, *R*_0_, *r*_*m*_, ECI and RGR all displayed a significant negative correlation with total phenolic content, total monomeric anthocyanin pigment content, DPPH of pomegranate cultivars; conversely, developmental time and RCR were positively correlated with these biochemical traits of pomegranate cultivars (Table 11).

## Discussion

Research on plant-herbivore interactions is one of the most important and multidisciplinary undertakings in plant biology, involving various disciplines to describe chemical and ecological processes influencing the outcome of plant-herbivore interactions (War et al., 2012). The results of the present study indicated that *E. ceratoniae* is able to complete its development, survive, and reproduce on all pomegranate cultivars examined; however, its growth rates significantly varied among the tested cultivars. The present research demonstrates the existence of interactions between biochemical characteristics of various pomegranate cultivars with growth rate, life table parameters, and nutritional indices of this pest. There are complex interactions of macronutrients with other dietary characteristics influencing lifespan and reproduction rate (Coskun et al., 2005; Chen et al., 2009). Primary plant metabolites, including nitrogen, protein, and carbohydrates have been shown to strongly affect the feeding and growth of insect herbivores (Soufbaf et al., 2012; Golizadeh and Abedi, 2017); however, less attention has been given to secondary plant metabolites and their effects on insect performance (Harvey, 2005; Ode, 2006; Mardani-Talaee et al., 2016).

In the present research, *E. ceratoniae* developed more slowly on Esfahani-Daneghermez and Malase-Danesyah cultivars, suggesting that the nutritional quality of these cultivars is less suitable for the feeding of larvae. It has been proposed that low nutritive quality of host diet is a possible measure offering resistance against insect herbivores (War et al., 2012; Akandeh et al., 2016). Variations in the duration of immature stages of *E. ceratoniae* might be attributed to differences in macronutrients and biochemical traits of the tested pomegranate cultivars. The shortest development time of *E. ceratoniae* on Shahvare-Danesefid cultivar may be explained by the higher carbohydrate value in this cultivar. Moreover, the secondary metabolites could be another factor affecting development time (Soufbaf et al., 2012; Nouri-Ganbalani et al., 2018). The content of major anthocyanin compounds and other secondary metabolites in Shahvare-Danesefid cultivar was lower than that of other tested pomegranate cultivars. There is a negative correlation between the development time of *E. ceratoniae* and macronutrients (especially carbohydrate) of pomegranate cultivars. Development time was positively correlated with major anthocyanin constituents, condensed tannins, total phenolic content, total monomeric anthocyanin pigment content, flavonoid, DPPH, and total titrable acidity of pomegranate cultivars, suggesting that pest development rate is negatively affected by respective metabolites and that these factors play a decisive role in the fitness of this insect. The longer development time of *E. ceratoniae* on Esfahani-Daneghermez cultivar may be due to higher levels of secondary metabolites n this cultivar. The range of larval and pupal periods of *E. ceratoniae* on tested cultivars is somewhat in agreement with the findings of Norouzi et al. (2008), Zare et al. (2013), and Mortazavi et al. (2016).

It has been reported that body weight is associated with the quality and quantity of food, which is one of the main biological indices of insect population dynamics (Li et al., 2004). The lower pupal weight of carob moths on Malase-Danesyah and Esfahani-Daneghermez cultivars indicated that the larvae fed on these cultivars did more poorly than on the other cultivars. The range of pupal weight for *E. ceratoniae* in the current study was greater than that reported by Mortazavi et al. (2016). The variations in nutritional quality or secondary compounds among the pomegranate cultivars can influence the larval and pupal development and can be reflected in the pupal weight of *E. ceratoniae*. The reduced body weight of this pest on cultivars Malase-Danesyah and Esfahani-Daneghermez might be attributed to the lower carbohydrate content and higher contents of secondary metabolites in these cultivars.

In this study, adults reared as larvae on the Shahvare-Danesefid and Toghe-Gardan cultivars presented higher fecundity and longevity, indicating suitability of these cultivars for reproduction of this pest. Regarding the larval and pupal weights, it is evident that the fecundity values can be correlated with the weights of larvae and pupae on the respective cultivars (Golizadeh and Abedi, 2016). This is illustrated by the present study, in which it was found that the development times of *E. ceratoniae* fed on pomegranate cultivars were positively correlated with levels of the major anthocyanin constituents and secondary compounds, suggesting that phytochemical metabolites play a negative role in the growth of this insect. In this study, the range of male and female longevities of *E. ceratoniae* was close to those reported earlier for *E. ceratoniae* reared on three pomegranate cultivars, including Malase-danesyah, Gabri, and Shahvar (Zare et al., 2013).

The results of the present investigation revealed that pomegranate cultivar is a major factor influencing life table parameters of *E. ceratoniae* so that its best and worst performances were observed on the Shahvare-Danesefid and Esfahani-Daneghermez cultivars, respectively. The reduced *R*_0_, *GRR*, *r*_*m*_ and λ of this pest on Esfahani-Daneghermez cultivar might be attributed to the positive correlation of these factors with primary metabolites as well as negative correlation with secondary metabolites of tested cultivars. Normally, higher *r*_*m*_ is related to shorter developmental time, lower mortality, and greater fecundity, which is true for *E. ceratoniae* reared on Shahvare-Danesefid. The range of *r*_*m*_ values of *E. ceratoniae* on various pomegranate cultivars tested in the present study was smaller than that reported by Norouzi et al. (2008), Zare et al. (2013), and Mortazavi et al. (2015). Such discrepancy might be attributable to either genetic differences in pest populations or variations in the experimental conditions and cultivars used for feeding of this pest.

The lower food consumption by *E. ceratoniae* on Esfahani-Daneghermez is likely attributable to the poor nutritional quality of this cultivar. The higher ECI value for *E. ceratoniae* on the Shahvare-Danesefid and Gorche-Tafti cultivars indicated that the larvae were able to convert digested food to body mass and that the weights of larvae improved on these cultivars. Furthermore, a significant correlation between ECI with starch and carbohydrate content indicated that larvae efficiently utilized carbohydrate for better growth. In contrast, the larvae reared on Esfahani-Daneghermez and Malase-Danesyah cultivars showed a lower ability to convert the ingested food into biomass, which led to the reduction in food consumption and weight of larvae (Nathan et al., 2005). The highest RCR value of *E. ceratoniae* on Esfahani-Daneghermez and Tabolarze-Mehrmahi could be due to the higher level of secondary compounds in these cultivars. Moreover, the variation in RGR and whole-body protein contents of populations reared on different pomegranate cultivars may have resulted from differences in primary plant metabolites among the cultivars.

In the present study, cluster analysis with respect to life table parameters, nutritional indices, and energy reserves of *E. ceratoniae* on tested pomegranate cultivars revealed two separate main clusters. Shahvare-Danesefid cultivar is the only member in cluster B as the most susceptible cultivar. The grouping within each class might be due to a high level of physiological similarity of pomegranate cultivars. Cluster A can be divided into two distinct sub-clusters (A1 and A2). Sub-cluster A2 consisted of Esfahani-Daneghermez and Malase-Danesyah (most relatively unsuitable cultivars), and other cultivars were grouped in sub-cluster A1 based on their similarity in primary and secondary metabolite contents (partially unsuitable cultivars).

## Conclusion

Results of the present study point to the apparent adaptability of *E. ceratoniae* to feeding on various pomegranate cultivars, albeit with different degrees of success. Our results indicate that insects fed on the Shahvare-Danesefid cultivar displayed the fastest development and highest adult fecundity and longevity, immature survival rate and nutritional performance, apparently as a consequence of the higher primary metabolite and lower secondary metabolite contents of the host plant. Therefore, this cultivar was the most suitable (least resistant) cultivar for *E. ceratoniae*, among those tested here. Moreover, the Esfahani-Daneghermez cultivar was the least suitable (most resistant) pomegranate cultivar for *E. ceratoniae*, apparently as a consequence of the higher secondary metabolite contents of the host plant. Consequently, Esfahani-Daneghermez was designated as the most unsuitable cultivar among those tested in the present work; we recommend that it be grown in areas where damage by *E. ceratoniae* is typically high, with the view to protecting or at least delaying infestation of pomegranate by this pest.

## Author Contributions

All authors read and approved the manuscript. This research is a part of Ph.D. thesis of ZA. AG as supervisor and ZA proposed the research subject and wrote the manuscript. ZA conducted the experiments. MS, MH, AJ-N, and H-RA participated in the data analysis and acted as advisors.

## Conflict of Interest

The authors declare that the research was conducted in the absence of any commercial or financial relationships that could be construed as a potential conflict of interest.
